# Simulating drifting fish aggregating device trajectories to identify potential interactions with endangered sea turtles

**DOI:** 10.1111/cobi.14295

**Published:** 2024-05-20

**Authors:** Lauriane Escalle, J. Scutt Phillips, J. Lopez, J. M. Lynch, H. Murua, S. J. Royer, Y. Swimmer, J. Murua, Alex Sen Gupta, V. Restrepo, G. Moreno

**Affiliations:** ^1^ Oceanic Fisheries Programme The Pacific Community (SPC) Nouméa New Caledonia; ^2^ Ecosystem and Bycatch Program Inter‐American Tropical Tuna Commission (IATTC) La Jolla California USA; ^3^ Center for Marine Debris Research (CMDR) Hawaii Pacific University (HPU) Waimanalo Hawaii USA; ^4^ Chemical Sciences Division National Institute of Standards and Technology (NIST) Waimanalo Hawaii USA; ^5^ International Seafood Sustainability Foundation (ISSF) Pittsburgh Pennsylvania USA; ^6^ The Ocean Cleanup Rotterdam The Netherlands; ^7^ NOAA Fisheries Pacific Islands Fisheries Science Center Honolulu Hawaii USA; ^8^ AZTI Tecnalia Sukarrieta Spain; ^9^ Climate Change Research Centre and ARC Centre of Excellence for Climate Extremes University of New South Wales Sydney New South Wales Australia

**Keywords:** ALDFG, bycatch, entanglement, fish aggregating devices, ghost fishing, Lagrangian models, Pacific Ocean, sea turtles, tropical tuna purse‐seine fishery, ALDFG, enredamiento, dispositivos de concentración de peces, modelos lagrangianos, Océano Pacífico, pesca accidental, pesca con red de cerco del atún tropical, pesca fantasma, tortugas marinas

## Abstract

Purse‐seine fishers using drifting fish aggregating devices (dFADs), mainly built with bamboo, plastic buoys, and plastic netting, to aggregate and catch tropical tuna, deploy 46,000–65,000 dFADs per year in the Pacific Ocean. Some of the major concerns associated with this widespread fishing device are potential entanglement of sea turtles and other marine fauna in dFAD netting; marine debris and pollution; and potential ecological damage via stranding on coral reefs, beaches, and other essential habitats for marine fauna. To assess and quantify the potential connectivity (number of dFADs deployed in an area and arriving in another area) between dFAD deployment areas and important oceanic or coastal habitat of critically endangered leatherback (*Dermochelys coriacea*) and hawksbill (*Eretmochelys imbricata*) sea turtles in the Pacific Ocean, we conducted passive‐drift Lagrangian experiments with simulated dFAD drift profiles and compared them with known important sea turtle areas. Up to 60% of dFADs from equatorial areas were arriving in essential sea turtle habitats. Connectivity was less when only areas where dFADs are currently deployed were used. Our simulations identified potential regions of dFAD interactions with migration and feeding habitats of the east Pacific leatherback turtle in the tropical southeastern Pacific Ocean; coastal habitats of leatherback and hawksbill in the western Pacific (e.g., archipelagic zones of Indonesia, Papua New Guinea, and Solomon Islands); and foraging habitat of leatherback in a large equatorial area south of Hawaii. Additional research is needed to estimate entanglements of sea turtles with dFADs at sea and to quantify the likely changes in connectivity and distribution of dFADs under new management measures, such as use of alternative nonentangling dFAD designs that biodegrade, or changes in deployment strategies, such as shifting locations.

## Abstract (es)

Simulación de las trayectorias de dispositivos de concentración de peces a la deriva para identificar las interacciones potenciales con las tortugas marinas en peligro de extinción

**Resumen:**

Los pescadores que usan redes de cerco con dispositivos de concentración de peces a la deriva (dFADs), hechos principalmente con bambú, boyas de plástico y redes de plástico, para concentrar y capturar atún, instalan entre 46,000 y 65,000 dFADs al año en el Océano Pacífico. Algunas de las problemáticas principales asociadas con este dispositivo de pesca de uso extenso son el enredamiento potencial de tortugas marinas y otras especies marinas en las redes de los dFADs; los desechos marinos y la contaminación; y el potencial daño ecológico por el varamiento en los arrecifes de coral, playas y otros hábitats esenciales para la fauna marina. Realizamos experimentos lagrangianos de deriva pasiva con la simulación de perfiles de deriva de los dFADs y los comparamos con áreas conocidas de importancia para las tortugas marinas. Esto fue con el objetivo de evaluar y cuantificar la conectividad potencial (número de dFADs instalados en un área que llegan a otra área) entre las áreas de instalación de dFADs y los hábitats oceánicos o costeros importantes para la tortuga laúd (*Dermochelys coriacea*) y la tortuga de carey (*Eretmochelys imbricata*), ambas en peligro crítico de extinción, en el Océano Pacífico. Hasta el 60% de los dFADs de las áreas ecuatoriales llegaron a los hábitats esenciales para las tortugas marinas. La conectividad fue menor sólo cuando se usaron áreas en donde actualmente hay dFADs instalados. Nuestras simulaciones identificaron regiones potenciales de interacción entre los dFADs y los hábitats de migración y alimentación de la tortuga laúd en el sureste tropical del Océano Pacífico; los hábitats costeros de ambas especies en el Pacífico occidental (p. ej.: zonas de archipiélagos en Indonesia, Papúa Nueva Guinea y en las Islas Salomón); y en el hábitat de forrajeo de la tortuga laúd en una gran área ecuatorial al sur de Hawái. Se requiere de mayor investigación para estimar el enredamiento de las tortugas marinas con los dFADs en el mar y para cuantificar los cambios probables en la conectividad y la distribución de los dFADs bajo nuevas medidas de manejo, como el uso alternativo de diseños que eviten el enredamiento y sean biodegradables, o cambios en las estrategias de instalación, como la reubicación.

## INTRODUCTION

The development of ecosystem‐based fishery management (Pikitch et al., 2004) has increased the awareness of fisheries’ impacts on ecosystems, including the incidental capture of vulnerable marine megafauna species (Lewison et al., 2014). Purse seines are the major gear type used to catch tropical tuna worldwide (Miyake et al., 2004; Williams & Ruaia, 2021). Their rate of bycatch is considered relatively low compared with other gear (Amandè et al., 2010; Kelleher, 2005). Tuna longline and gillnet fisheries have megafauna (e.g., sharks, rays, marine mammals, and sea turtles) discard rates 4–5 times greater than tuna purse seiners (Dagorn et al., 2013; Kelleher, 2005; Peatman et al., 2018; Swimmer et al., 2020). The tropical tuna purse‐seine fishery has two principal fishing modes with different levels of bycatch and environmental impacts: fishing on free‐swimming schools and fishing on schools associated with floating objects (Dagorn et al., 2013; Hall, 1998). The latter is made on natural logs, marine debris, or human‐made fish aggregating devices (FADs) and represents large proportions of the recent purse‐seine tuna catch in all oceans, including the Pacific Ocean (over 40% of the catch) (IATTC, 2021a; Williams & Ruaia, 2021). In general, drifting fish aggregating devices (dFADs) in the Pacific are bamboo rafts with floats wrapped in polyester or nylon netting and submerged appendages of synthetic netting reaching 50‐m depth on average (Escalle, et al., 2022; Lopez et al., 2019). Satellite echosounder buoys are attached to dFADs for geolocation and remote estimation of tuna biomass beneath (Lopez et al., 2014). It is estimated that up to 65,000 dFADs are deployed annually in the Pacific Ocean (Escalle, et al., 2021; Lopez et al., 2020), which has raised some environmental concerns (Leroy et al., 2013). First, higher catch rates of juvenile tuna raised concerns related to the sustainability of tuna stocks (Dagorn et al., 2013). Second, dFAD bycatch rates, including marine megafauna (sharks and sea turtles), are higher than in other purse‐seine fishing modes (Bourjea et al., 2014; Dagorn et al., 2013). Third, marine megafauna entanglement (so‐called ghost fishing) can occur on dFAD's netting. Finally, the loss or abandonment of dFADs by fishers can lead to marine and coastal debris and pollution and structural damage to fragile ecosystems, such as coral reefs (Balderson & Martin, 2015; Escalle et al., 2019; Maufroy et al., 2015). Management measures implemented to mitigate the impacts of dFADs include seasonal and area closures, prohibiting the use of netting in dFAD construction, transitioning to biodegradable dFADs, limiting the number of dFAD that can be monitored, and encouraging dFAD retrieval (Pons et al., 2023).

Interactions with fishing gear are among the major threats to sea turtle populations worldwide (Wallace et al., 2013). However, tropical tuna purse‐seine fishery impacts have previously been considered low (Bourjea et al., 2014; Montero et al., 2016; Swimmer et al., 2020), largely as a result of low bycatch rates and direct interaction resulting in very few mortalities (IATTC, 2021b; Peatman et al., 2018). In the Pacific Ocean, 100% observer coverage is required for all large purse‐seine vessels (Hall & Roman, 2013; Panizza et al., 2021), allowing for accurate estimates of sea turtle bycatch. Although all 5 species of sea turtles present in the Pacific Ocean are found as bycatch in purse‐seine fisheries, olive ridley (*Lepidochelys olivacea*), green (*Chelonia mydas*), and loggerhead (*Caretta caretta*) sea turtles are accidentally caught in higher proportion. Hawksbill (*Eretmochelys imbricata*) and leatherback (*Dermochelys coriacea*) turtles are rarely captured, possibly and partly because of their low population numbers; hawksbills globally and both Pacific leatherback populations are considered critically endangered (Mortimer & Donnelly, 2008; Wallace et al., 2013). Thus, we focused on highly or critically endangered species that are underrepresented in observer data, likely due to depleted status, low abundance, and low direct interaction rates with the purse‐seine fishery but that could be highly vulnerable to dFAD interaction through entanglement or habitat damage.

Leatherback turtle populations have dramatically declined in the Pacific Ocean at rates of ∼5% annually since the 1980s, with fisheries interactions playing a major role (Bailey, Benson, et al., 2012; Benson et al., 2020; Wallace et al., 2013). There are 2 distinct populations of leatherback turtles in the Pacific Ocean. The western Pacific population nests mainly in Indonesia, Papua New Guinea (PNG), and Solomon Islands, including the last sizable nesting population in the entire Pacific in Papua Barat, Indonesia (Tapilatu, 2014). The movements and locations of juvenile leatherbacks are not well known, but simulations of their active dispersal suggest two dispersal habitats from Indonesia: a large oceanic region from 180°W to the Americas and between 25°N and 40°N and along the coast of Mexico (Gaspar & Lalire, 2017). Adults have been observed migrating across the Pacific to forage on jellyfish off the coast of central California (Benson et al., 2011). Two additional foraging habitats for adults of this population are the Kuroshio Extension and a zone in the northern equatorial Eastern Pacific Ocean (EPO) (Benson et al., 2011). The EPO population nests from Baja California Sur to northern Ecuador, mostly in Mexico, Costa Rica, and Nicaragua (Bailey, et al., 2012; Laúd opo Network, 2020; NMFS & FWS, 2020; Shillinger et al., 2008, 2011). Adults migrate to forage off the coasts of Peru and Chile and have migratory corridors through Colombian and Ecuadorian waters (Bailey et al., 2012; Laúd opo Network, 2020; Shillinger et al., 2008, 2011).

Hawksbill sea turtle populations have declined >80% globally since the beginning of the 20th century, predominantly due to the trade of its shell (Mortimer & Donnelly, 2008). In the Pacific, there are several small rookeries (typically <100 nests/year) throughout the tropical Pacific, including Costa Rica and the main Hawaiian Islands (MHI). The majority of nesting occurs in Solomon Islands (<1000 nests/year) (SWOT, 2008). Hawksbills forage primarily on sponges on coral reefs relatively close to nesting grounds, so they do not typically make large migrations (Gaos et al., 2020; SWOT, 2008).

The extensive use of dFADs in the fishery is concerning for these endangered populations of sea turtles, given the potential for entanglement in their netting (Filmalter et al., 2013). Despite the high observer coverage, interactions largely remain unobserved because dFADs may be visited only once or twice and on‐board fisheries observers generally cannot detect sea turtles entangled in the submerged appendages. Moreover, once lost or abandoned, dFADs may still entangle sea turtles when drifting unmonitored. Areas of overlap between dFAD aggregations and important sea turtle migratory routes or foraging areas could therefore highlight potential high‐risk areas and inform potential mitigation measures. In addition, dFADs stranded in shorelines and nearshore habitats could have indirect impacts on critical nesting or coral reef habitats for sea turtles. Although no one has investigated the specific impact of stranded dFADs on sea turtle nesting, other large debris on nesting beaches has been shown to deter sea turtles from nesting (Fujisaki & Lamont, 2016; Laurance et al., 2008).

We assessed potential dFAD interactions with critically endangered leatherback and hawksbill sea turtles, focusing on entanglement and critical habitat damage impacts. Large numbers of dFAD observations are required to robustly examine factors affecting high dFAD aggregation at sea or in coastal regions and their interaction with sea turtles. Although highly informative, access to real dFAD trajectories is currently limited in the Pacific Ocean (Escalle, et al., 2021; Lopez et al., 2020), particularly in areas outside the main purse‐seine fishing grounds (10°N to 10°S). The dFAD trajectory data are owned by the fishing companies deploying, monitoring, and tracking these devices. Although some tuna Regional Fisheries Management Organizations (RFMOs) require access to this information, historic information and complete data on dFADs’ entire trajectories are not readily available because fishers typically deactivate the buoys tracking dFADs when the devices leave the fishing grounds (Pons et al., 2023). Previous analyses of dFAD stranding events in the Pacific show that the number of observed stranded dFADs is too small to establish a reliable distribution pattern (Escalle et al., 2019). In this context, Lagrangian simulation is a useful tool to help establish spatial and temporal patterns based on much larger numbers of virtual dFADs (Escalle et al., 2019; Imzilen et al., 2018; Scutt Phillips et al., 2019). However, the simulation is informed, when possible, with real dFAD data, such as areas of real dFADs deployments.

Similarly, insights from fishers themselves can provide important information to complement, validate, or guide scientific analyses (Moreno, et al., 2007). We aimed to examine simulated dFAD trajectories to identify likely dFAD aggregation and stranding areas, especially those that overlap with important habitats for critically endangered leatherback and hawksbill sea turtles. We focused on at‐sea entanglement risk for leatherbacks and coastal habitat damage for foraging hawksbills and nesting turtles of both species. Our overarching objective was to inform management of the dFAD fishery in the Pacific to limit the adverse effects it might cause on sea turtles and their critical habitats. Moreover, it would help inform the magnitude of these impacts in comparison with other anthropogenic threats (e.g., other fisheries, coastal development, pollution, climate change) to address and mitigate the primary risks.

## METHODS

Lagrangian simulations were carried out to examine the drift of particles, representing virtual fish aggregating devices (vFADs), in the Pacific Ocean (Escalle et al., 2019; Scutt Phillips et al., 2019). These simulations tracked the trajectory in space and time of simulated particles advected by ocean currents with output from an ocean general circulation model. The objective was to determine the probability and percentage of dFADs arriving at key sea turtle habitats over time scales comparable to current dFADs use, including their full lifetime at sea before reaching coastal or specific oceanic areas.

### Study area

**FIGURE 1:**
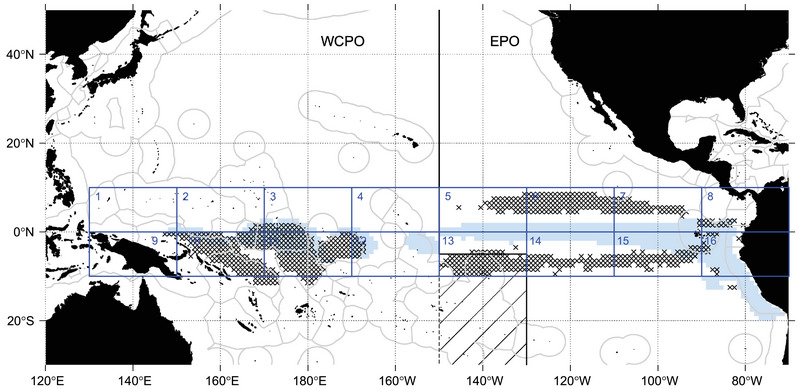
Spatial distribution of the release areas of virtual drifting fish aggregating devices (dFADs) throughout the equatorial Pacific in the drift trajectory simulations, including the equatorial zones (EZs) used in scenario 1 (dark blue rectangles 1–16) and the dFAD zones (FZs) used in scenario 2 corresponding to 1° cells included in the main dFAD deployments areas (light blue cells) and main dFAD densities areas (black crosses) (black line, boundary between the WCPFC [Western and Central Pacific Fisheries Commission] and IATTC [Inter‐American Tropical Tuna Commission] convention areas; hatched region, overlapping areas of the conventions).

**FIGURE 2:**
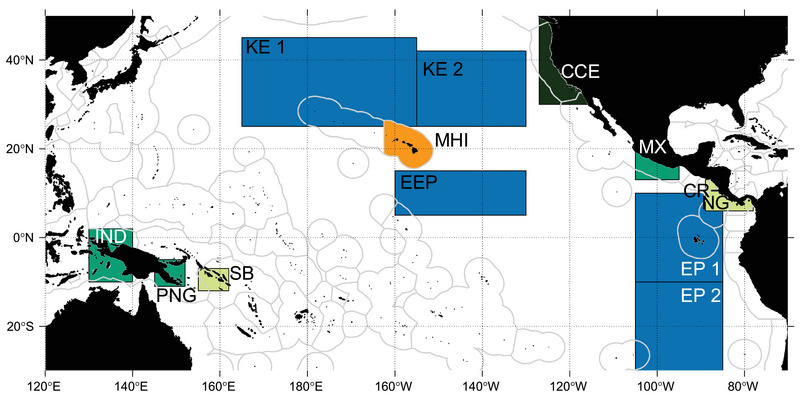
Spatial distribution of sea turtle habitat (turtle zones [TZs]) used in the simulations of drifting fish aggregating devices trajectories and corresponding to important oceanic areas (blue) for leatherback turtles and coastal areas for leatherback foraging (dark green), leatherback nesting and foraging (medium green, except for Mexico [MX], nesting only), hawksbill nesting and foraging (orange), and leatherback and hawksbill nesting and foraging (light green) (KE, Kuroshio Extension; EEP, Equatorial Eastern Pacific; CCE, California Current Ecosystem; IND, Indonesia; MHI, main Hawaiian Islands; PNG, Papua New Guinea; SB, Solomon Islands; CR‐NG, Costa Rica–Nicaragua; EP, Eastern Pacific).

Simulations covered the Pacific Ocean (120°E−90°W and 50°N−30°S), including key sea turtle habitats (Figure 1). We examined vFAD drift trajectories that connected dFAD deployment areas (Figure 1) and essential habitats in leatherback and hawksbill sea turtle life histories (Figure 2).

Two types of deployment areas were used: the entire tropical equatorial zone (EZ) from 10°S to 10°N divided into 16 large rectangular cells of 20° longitude × 10° latitude (EZ) and areas where dFAD deployment and high density are known (dFAD zones [FZs]) (Figure 1). High‐dFAD‐density and high‐dFAD‐deployment areas were derived from monthly average number of active satellite buoys and the annual average number of deployments. These data came from Escalle, et al. (2021) (Parties to the Nauru Agreement dFAD tracking database, 2016−2020) for the Western and Central Pacific Ocean (WCPO) and the Inter‐American Tropical Tuna Commission buoy (2018−2020) and observer (2016−2020) databases for the EPO (Lopez et al., 2020). Cells corresponding to values of density and deployments above the 90th percentile were selected to simulate deployments for each convention area separately (Figure 1).

The interactive Ocean Biodiversity Information System Spatial Ecological Analysis of Megavertebrate Populations (OBIS‐SEAMAP) platform was used to establish sea turtle habitat zones (TZs) (Figure 2). These data, curated by the State of the World's Sea Turtles (SWOT), include nesting and habitat distribution maps of leatherback and hawksbill sea turtles (https://seamap.env.duke.edu/swot). Scientific publications and expert opinions were also used to define critical habitat areas for the 2 species. For leatherback sea turtles, large spatial boxes (green in Figure 2) were assigned for primary critical coastal nesting and foraging habitats (Bailey, et al., 2012; Benson et al., 2011; Laúd opo Network, 2020; NOAA Fisheries, 2022a). Oceanic habitats (blue in Figure 2) were also identified as key leatherback foraging areas along their migration routes (Benson et al., 2011; Shillinger et al., 2011). Because hawksbills spend less time in pelagic waters, the focus was on their coastal nesting and foraging habitats (light green and orange in Figure 2), which overlap with leatherback nesting areas in Costa Rica and Solomon Islands (SWOT, 2008). The selected TZs, based on the above available information, do not represent the entire habitat distribution of these species and may not contain all important habitat. For example, leatherback turtles inhabit the region toward the coast of South America, partially included in the TZs Eastern Pacific 1 and 2 (EP1 and EP2) (Degenford et al., 2021), and forage near southeastern Australia and northern New Zealand outside the latitudinal boundaries of our study (Benson et al., 2011).

### Lagrangian simulations and connectivity

Lagrangian simulations were implemented using the Parcels framework (Delandmeter & van Sebille, 2019), and two scenarios were investigated. Passively drifting Lagrangian particles representing vFADs were released with a random position across the seeding areas defined in each scenario (Appendix S1).

In scenario 1, particles were released from the EZs (Figure 1) to examine the connectivity of the entire equatorial fishing ground with sea turtle habitats. This idealized scenario encompasses the whole tropical Pacific, where dFADs are commonly monitored. Approximately 6000 randomly distributed particles across each EZ box were released once per week for one year and tracked for 24 months.

In scenario 2, particles were released from the FZs (Figure 1). This scenario focused on the current fishing strategy and, hence, takes advantage of areas where dFADs are currently deployed (scenario 2a) and aggregate (scenario 2b). Approximately 6000 randomly distributed particles across each FZs were released once per week for one year and tracked for 24 months.

In both scenarios, particles were released on 1 July, corresponding to the beginning of dFAD‐closure periods in the Pacific Ocean and the start of the greatest oceanographic effects of each El Niño–Southern Oscillation (ENSO) phase. Each scenario was repeated three times to test the sensitivity of our results to oceanographic conditions: 1 July 2012 (ENSO neutral), 1 July 2010 (moderate La Niña), and 1 July 2015 (strong El Niño). Due to the similarity in results across ENSO periods, results from all sensitivity experiments were merged by particle drift time in the figures and table. Approximately 93,000 particles (∼30 particles/100 km^2^) were seeded each week for scenario 1, and approximately 51,000 were seeded per week for scenario 2. In total, over 8 million particles were used to sample the space–time domain of our simulation experiments.

Particles were simulated with a dFAD‐type drift profile by integrating the top 50‐m current velocities (median dFAD net depth of 40 and 50 m in the EPO and WCPO, respectively [Escalle et al., 2017; Lopez et al., 2020]). Zonal and meridional ocean current data at 0.1° grid resolution were taken from the eddy resolving Bluelink Reanalysis 2020 ocean circulation model (BRAN 2020, Chamberlain et al., 2021), which combines multiyear observations of ocean data to provide historical estimates of ocean state in space and time. Given the current uncertainty of subgrid‐scale processes below the resolution of this model, such as stokes drift and windage on drogued dFAD drift, no diffusion or processes other than advection by currents were included. The vFADs were advected using a fourth‐order Runge–Kutta interpolation scheme with a 6‐h time step, and their positions were archived at a weekly time step.

The particle drift trajectories were then used to calculate the spatial distribution of vFADs after various drift durations, alongside metrics of potential connectivity. vFAD density maps summarized the integrated distribution of vFADs across a particular time of the experiment. Connectivity matrices were used to summarize the connectivity of different zones by comparing the trajectories of individual vFADs over time, quantifying the proportion of vFADs released in one equatorial fishing ground deployment zone (EZ or FZ) and arriving at a TZ for each drift time. Drift times were summarized as short (˂3 months), moderate (3−12 months), and long (12−24 months). By calculating such proportional movement rates, these matrices should be interpreted as the probability of movement (i.e., connectivity) between the 2 zones, given the assumptions of the physical ocean model and each drift time. Simulation output data used for the connectivity matrices and density maps are available at https://zenodo.org/doi/10.5281/zenodo.10815559.

### Local fishers’ knowledge

To contrast the results of the simulation and vFAD drift patterns with fishers’ observations of real dFADs, three workshops were conducted with tuna purse‐seine fishers who had decades of experience working with dFADs in the Pacific Ocean. Workshops were conducted in the country of origin of these fishers (Croatia, Spain, and Ecuador) in 2022. In total, 13 fishers from Spain operating in both the EPO and WCPO, four fishers from Croatia operating in the WCPO, and 30 fishers from Ecuador operating in the EPO were consulted. During the meetings, fishers were asked to create maps indicating typical dFAD trajectories in their areas of operation, locations of sea turtle sightings, and areas where they had observed interactions with sea turtles (all species combined) during fishing operations. Maps were then compared with results from simulations to engage in discussions with fishers to assess whether the simulation results regarding dFAD circulation, accumulation at sea, and connectivity with specific turtle areas aligned with their empirical knowledge. The organizers of this workshop adhered to a set of ethical principles to ensure the integrity and well‐being of human participants. These principles encompassed obtaining informed consent from all participants, maintaining confidentiality and privacy, fostering an environment of respect for diverse perspectives, and ensuring fair treatment and equity. By upholding these ethical standards, the authors sought to create a safe and inclusive space for intellectual exchange and collaboration.

## RESULTS

### Simulations

**FIGURE 3:**
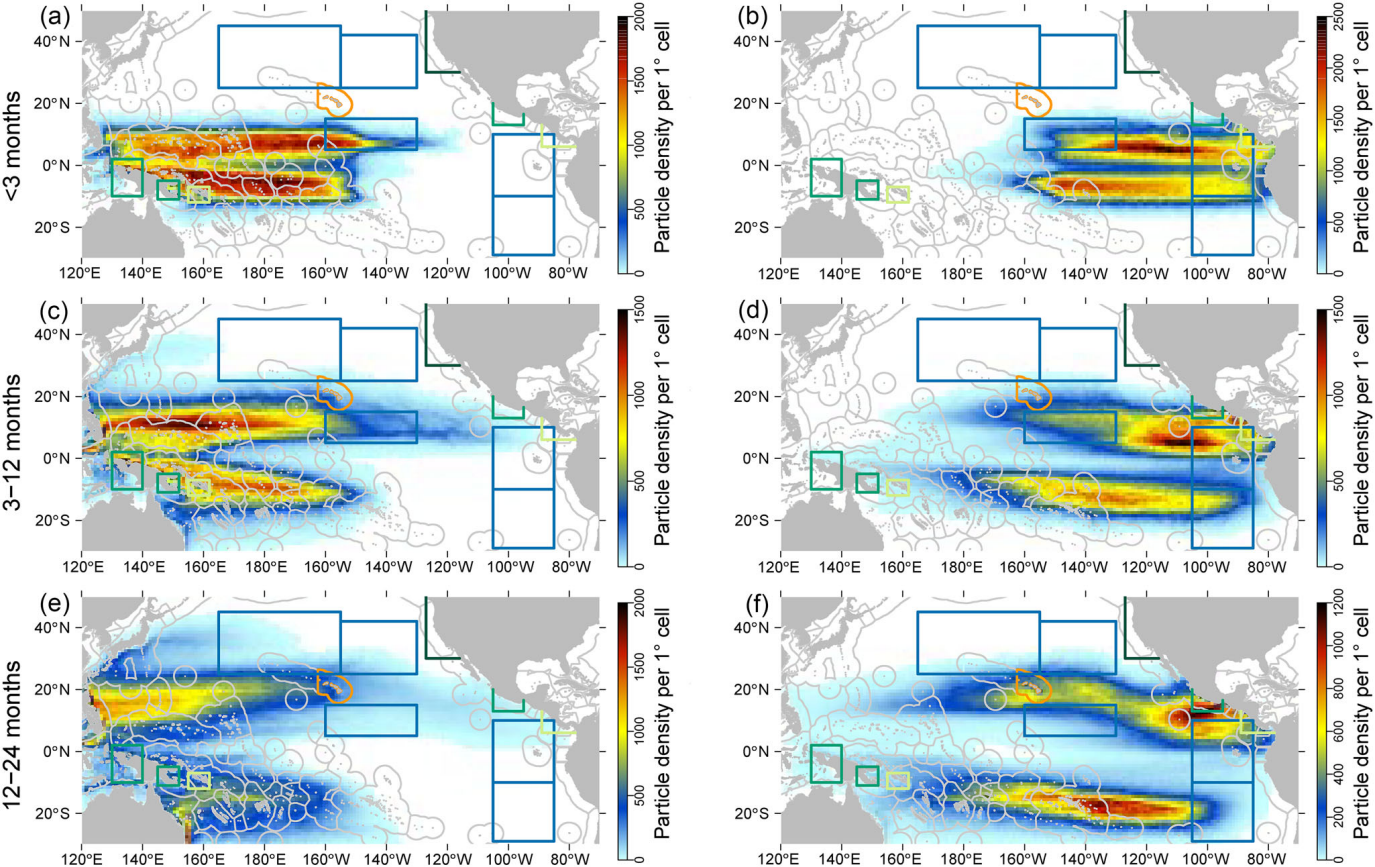
Time‐integrated spatial probability density for virtual fish aggregating device particles deployed, evenly across the equatorial region (scenario 1) in the (a, c, e) Western and Central Pacific Ocean (equatorial zones 1−4 and 9−12) and the (b, d, f) Eastern Pacific Ocean (equatorial zones 5−8 and 13−16) during the three combined El Niño–Southern Oscillation periods considered and over three drifting periods after deployment (blue rectangles, oceanic areas for leatherback turtles; dark green rectangle, coastal areas for leatherback foraging; medium green rectangles, leatherback nesting and foraging area; orange polygon, hawksbill nesting and foraging area; light green rectangle, leatherback and hawksbill nesting and foraging).

**FIGURE 4:**
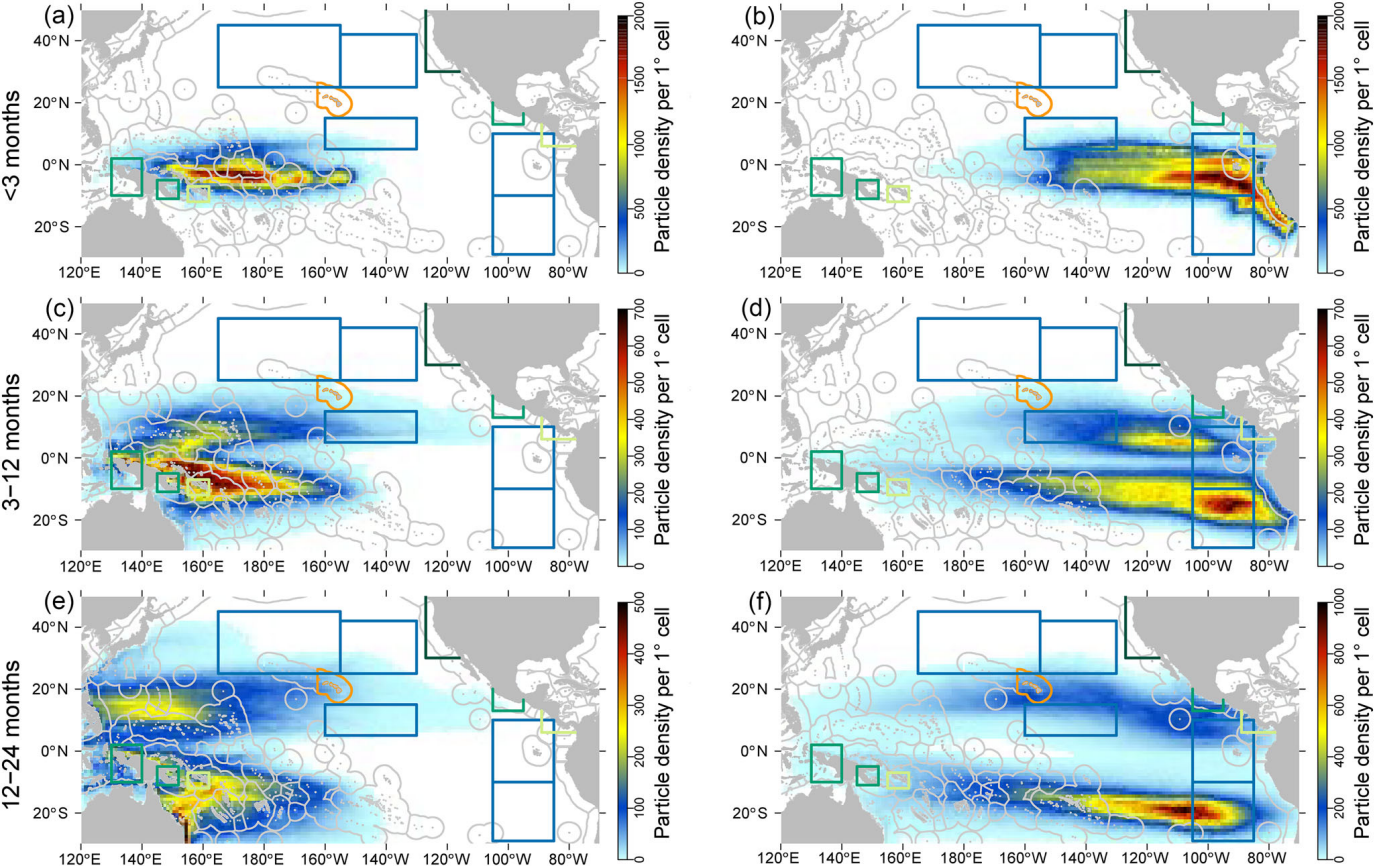
Time‐integrated spatial probability density for virtual fish aggregating device particles deployed evenly across dFAD deployment areas (scenario 2a) in the Western and Central Pacific Ocean (equatorial zones 1−4 and 9−12) and the (b, d, f) Eastern Pacific Ocean (equatorial zones 5−8 and 13−16) during the three combined El Niño–Southern Oscillation periods considered and over three drifting periods after deployment (blue rectangles, oceanic areas for leatherback turtles; dark green rectangle, coastal areas for leatherback foraging; medium green rectangles, leatherback nesting and foraging area; orange polygon, hawksbill nesting and foraging area; light green rectangle, leatherback and hawksbill nesting and foraging).

**FIGURE 5:**
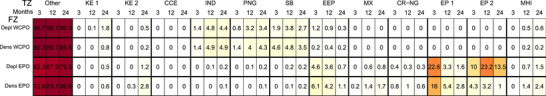
Percent connectivity matrix of virtual fish aggregating device (vFAD) particles in the simulation during the three El Niño–Southern Oscillation periods considered combined from scenario 2 in which vFADs were seeded in known dFAD zones (FZs; Depl, high‐deployment area; Dens, high‐density area [rows] within 3, 12, or 24 months [subcolumns]) (cell colors, proportion of simulated particles arriving in each turtle zone by drift time increases with increasing intensity; other, locations outside the specified turtle zone; WCPO, Western and Central Pacific Ocean; EPO, Eastern Pacific Ocean).

Overall, simulations suggested strong connectivity (vFAD arriving in one TZ above 10% of vFADs deployed in one FZ) between the equatorial FZ and the majority of the large sea TZs when vFADs were deployed evenly across EZs (scenario 1) (Figure 3; Appendix S2). A summary of the maximum percentage of connectivity for each TZ shows that the greatest connectivity of vFAD origin was with Eastern Pacific 1 and 2 (EP 1 and EP2) in both scenarios (Table 1). The leatherback migrating and foraging areas in the Eastern Equatorial Pacific (EEP) had the next greatest connectivity with the equatorial area, only slightly greater than Indonesia (IND), Solomon Islands (SB), and PNG nesting sites. The eastern leatherback and hawksbill nesting sites (MHI, Mexico [MX], Costa Rica–Nicaragua [CR‐NG], and Kuroshio extensions 1 and 2 [KE1 and KE2]) had lower connectivity with vFADs, and the California Current ecosystem (CCE) foraging site had no connectivity with vFADs. However, when considering only those vFADs seeded in areas of known dFAD deployment and high density (scenario 2), the connectivity was significantly reduced (Figures 4 & 5; Table 1). Areas of high dthreeFAD deployment (scenario 2a) and high dFAD density (scenario 2b) were associated with substantial proportions of vFADs reaching the large EP1 and EP2 foraging zones (up to 23.2%) and the EEP between 5°N and 15°N (up to 6.1%), known as important foraging habitats for leatherback turtles, although vFADs were transiting rather than accumulating (Figures 4 & 5; Appendix S3).

**TABLE 1:** The maximum percentage of virtual fish aggregating device (vFAD) connectivity (i.e., percentage of vFADs deployed in one area and found in another area after a certain drift period) with each sea turtle habitat zone (turtle zone) (data in Figure 5 and Appendix S2).

				vFAD^b^ arrival into turtle zone in scenario 1				vFAD^b^ arrival into turtle zone in scenario 2		
Zone^a^	Species	Leatherback population	Behavior	Max. %	Origin EZ	Origin region	Drift months	Max. %	Origin FZ	Drift months
EP2	Dc	Eastern	Foraging and migrating	57.9	16	EPO	12	23.2	Depl EPO	12
EP1	Dc	Eastern	Foraging and migrating	52.1	16	EPO	3	22.6	Depl EPO	3
EEP	Dc	Western	Foraging and migrating	50	5	EPO	3	6.1	Dens EPO	3
IND	Dc	Western	Nesting and foraging	26.2	9	WCPO	3	4.9	Dens WCPO	12, 24
SB	Dc and Ei	Western	Nesting and foraging	10.6	10	WCPO	3	4.6	Dens WCPO	3
PNG	Dc	Western	Nesting and foraging	7.4	10	WCPO	24	4.3	Dens WCPO	24
KE2	Dc	Western	Foraging and migrating	9.8	5	EPO	24	2.8	Dens EPO	24
MHI	Ei	–	Nesting and foraging	9.6	5	EPO	12	2.4	Dens EPO	24
KE1	Dc	Western	Foraging and migrating	8.3	4	WCPO	24	1.8	Depl WCPO	24
MX	Dc	Eastern	Nesting	10.2	8	EPO	12	1.7	Dens EPO	24
CR‐NG	Dc and Ei	Eastern	Nesting (both), foraging (Ei)	22.5	8	EPO	3	0.8	Dens EPO	3
CCE	Dc	Western	Foraging	0	–	–	–	0	–	–

Abbreviations: Dc, leatherback; Dens, density; Depl, deployment; Ei, hawksbill sea turtles; EPO, Eastern Pacific Ocean; EZ, equatorial zone; FZ, dFAD zone; WCPO, Western and Central Pacific Ocean.

^a^ Turtle zones (TZs) are shown in Figure 2.

^b^ Origin zones (EZs) are shown in Figure 1.

In the WCPO, the small coastal sea turtle nesting habitats in PNG and SB consistently received and retained vFADs arriving from the southern equatorial regions of the WCPO in scenario 1 (up to 7% and 10%) (Figure 3; Appendix S2). This was also the case, despite connectivity being lower (up to 5%), in scenario 2 (Figures 4 & 5; Appendix S3). The archipelagic IND nesting habitat had similarly high connectivity with vFADs arriving from mostly one region of the WCPO in scenario 1, the southwestern EZ 9 (26%); however, the relatively low densities in this region suggest that although vFADs reached this region, they did not remain there for extended periods (Figure 3; Appendix S2). Again, a similar pattern with weak connectivity was seen between IND and EZ 9 in scenario 2 (up to 5% after 12 months of drift) (Figures 4 & 5).

In the EPO, central and south regions of the equator, two large zones important for the migration and foraging of leatherback turtles (EP1 and EP2), had very high levels of connectivity with vFADs originating from the whole equatorial EPO region (scenario 1), both north and south of the equator (up to 57.9% of all deployed vFADs) (Figure 3; Appendix S2). This connectivity was lower in scenario 2 (up to 23.2%) (Figures 4 & 5). The majority of vFADs transited through these zones of sea turtle migration (EP1 and EP2) into a gyre of accumulation in the southeastern Pacific Ocean, which only partially overlapped with these TZs (Figure 3; Appendix S2). Similarly, the regions important for eastern Pacific leatherback nesting in MX and CR‐NC were moderately connected with vFADs deployed near the equator in scenario 1 (up to 22%) (Figure 3; Appendix S2). Again, this was greatly reduced when considering vFADs deployed under scenario 2 (up to 2%) (Figures 4 & 5; Appendix S3).

The vFADs arrived in the KE1 and KE2, where western Pacific leatherback turtles forage and migrate (Bailey, H., Benson, et al., 2012; Benson et al., 2011), via the Kuroshio current or more directly. The vFADs arriving in these regions mostly had drift times of at least 1 year and originated from EZs in the northern WCPO and northwestern EPO in scenario 1 (up to 10%) (Figure 3; Appendix S2). Once again, the magnitude of this connectivity was considerably lower in scenario 2 (up to 2.8% from the EPO and 1.8% from the WCPO) (Figures 4 & 5; Appendix S3).

The EEP oceanic region was identified as a highly transited corridor for western Pacific leatherback turtles migrating to the CCE foraging habitat (Benson et al., 2011). Connectivity was strong between the EEP and EZ 3−6 (up to 50%) in the first 3 months of scenario 1, and these origins were nearly equally split between EPO and WCPO (Figure 3; Appendix S2). This was expected because the EEP partially overlapped with EZ 4 and 5 and was adjacent to EZ 3 and 6 (Figures 1 & 2). In the more realistic scenario 2, up to 6.1% of the vFADs from high‐dFAD‐density areas in the EPO arrived in the EEP after 6 months, compared with up to 1.2% from the WCPO deployment areas (Figures 4 & 5; Appendix S3). No vFADs arrived in the CCE region in either scenario (Figure 5; Appendix S2).

### Local fisher knowledge

**FIGURE 6:**
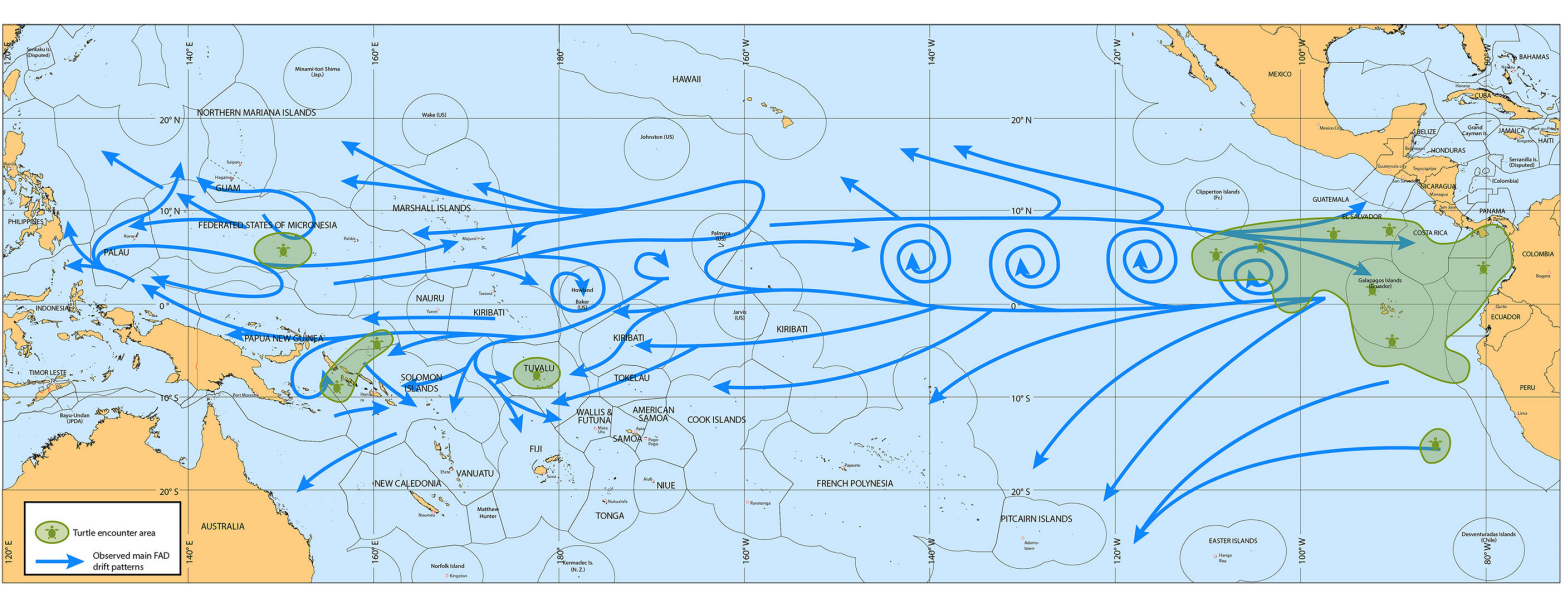
Main drifting fish aggregating device (dFAD) drift patterns (blue arrows) and areas of purse seiner and sea turtle interactions (green polygons) based on knowledge shared by purse‐seine fishers fishing in the Western and Central Pacific Ocean and the Eastern Pacific Ocean. Maps from all three fisher workshops were combined in this map of the basin. Map produced by B. Colas.

Fishers were confident in their understanding of dFAD trajectories in their fishing grounds, and they had a general idea of which direction unmonitored dFADs (i.e., when geolocation buoys are deactivated) might drift when they exit their fishing grounds. The areas of interactions described by fishers were broadly similar to the ones we identified in the literature and with expert opinion (Figures 2 & 6). This included a large interaction area in the equatorial and southern EPO that covered EP 1 and CR‐NC, although it extended to 110°W and the PNG and SB leatherback and hawksbill nesting sites (Figure 6). Fishers also highlighted Tuvalu and the central part of the Federated States of Micronesia as other sea turtle interaction areas.

In terms of drift, fishers described dFAD patterns in their main fishing grounds, with fishers operating within the same areas describing similar drift patterns (Figure 6). Fishers in the EPO described patterns similar to those predicted by the simulations, particularly the large tendency of dFADs that veer southwest of the equator toward the southeastern Pacific area (e.g., French Polynesian Islands) (Figure 6), and northwest toward the Hawaiian Islands. The EPO fishers also described the tracks of dFADs when they drift north of the equator, showing dFADs becoming entrained in gyres, a limited number of dFADs moving toward the Central and South American coast, and some exiting these gyres and drifting northwest toward Hawaii. In contrast, some of the longer lasting dFADs that follow the equatorial currents may enter WCPO waters, at which point most EPO fishers deactivate the dFAD tracking buoys or transfer them to WCPO fishers. For the fishers operating in the WCPO, there was a consensus that dFADs track westward with many ending up near islands, such as PNG and SB. Fishers operating in the WCPO also highlighted the difficulty of predicting dFAD movements in this region because there can be high, small‐scale variability in currents due to seasonal and inter annual effects.

## DISCUSSION

We identified potential origin areas of dFADs that arrive in important sea turtle habitats by employing Lagrangian passive drift simulations to estimate the potential dFAD densities and connectivity in these zones. Simulation results were supported by local fishers’ knowledge gained through decades of dFAD drift observations and at‐sea turtle interactions in the EZ.

### Simulation experiments

The dynamics of our simulation experiment results compared generally well with the description of observed dFAD drifts made by purse‐seine fishers at large scales. Nevertheless, the connectivity predicted by the model rests on several assumptions. Most importantly, our drift model with passive advection under ocean flow in the top 50 m of the water column did not include more complex processes that can affect the trajectory of floating objects, such as windage or stokes drift (Van Sebille et al., 2019). Surface velocity components of drogued dFADs are highly correlated with oceanic drifters, used by oceanographers to measure ocean currents (Imzilen et al., 2018). Moreover, a comparison of similar passive drift simulations with observed dFAD densities shows generally good correlations at large scales in the WCPO (Scutt Phillips et al., 2019), which were not present when more wind‐driven, surface‐only currents were used to drive movement. Thus, it is recommended that our simulation patterns, which generally concur with fishers’ knowledge, are validated using observed dFAD trajectories (Imzilen et al., 2018), for which there is a limited, but growing, database (Lopez et al., 2023).

Such a validation with observed dFAD trajectories is also needed beyond the equatorial fishing grounds where fishers have knowledge and where several of the sea turtle habitats explored in this study are located. Other processes affecting dFAD drift may dominate connectivity once they depart from the directed flow of the equatorial area. We assumed a constant 50‐m drift profile throughout the simulated life of all dFADs, where in reality dFADs have a range of depths and designs (Escalle, et al., 2023), and many observed stranded dFADs have lost submerged appendages, often leaving only the rafts or just the satellite buoys (Donohue, 2005; Royer et al., 2023). The connectivity and overlap with TZs caused by this degradation could be explored by allowing particles to be affected by shallower ocean currents and different lifetimes.

### Key dFAD dynamics and potential sea turtle impacts

Under scenario 1, considerable connectivity was found in simulations between many TZs and the equatorial Pacific dFAD fishing area. More realistic dFAD deployment and density are likely to vary considerably at scales of months to years. We have represented this by evenly deploying vFADs across areas previously identified as containing high dFAD density and numbers of deployments under scenario 2. These results showed that, given current information on where purse‐seine fleets deploy and use dFADs, potential connectivity between the equatorial fishing ground and important turtle habitats appears reduced in most cases. However, to predict the actual impact of dFADs on turtle populations, estimating the actual level of interaction and fate of sea turtles that interact with dFAD structures in the area of overlap identified here is critical.

For dFADs deployed in the WCPO, it appears that the main areas of concern are the nesting and foraging habitats of Indonesia, PNG, and Solomon Islands. The vFADs appeared more transient through the Indonesia zone arriving after moderate drift times, whereas PNG and Solomon Islands consistently received and retained vFADs arriving from the southern equatorial regions of the WCPO. Although similar patterns were detected under both scenarios, reduced connectivity was detected when observed dFAD deployment and high dFAD density were used to seed vFADs (i.e., maximum of 5% connectivity in scenario 2 vs. 26% in scenario 1). In addition, 2 critical migratory and foraging regions of the western Pacific leatherback population (Equatorial Eastern Pacific and Kuroshio Extensions 2) showed moderate and low connectivity to vFADs from both the WCPO and the EPO (6% and 3%, respectively).

For dFADs deployed in the EPO, the main areas of concern appear to be the oceanic leatherback turtle migration and feeding grounds in the southeastern tropical Pacific Ocean (Eastern Pacific 1 and 2; up to 23% in scenario 2 and 58% in scenario 1), which experience dense aggregation of dFADs deployed in the EPO over short to moderate drift durations (0−12 months). Given the lack of inhabited land mass in this area, aside from the Pitcairn Islands, a large potential aggregation of dFADs leaving the main purse‐seine fishing ground may currently exist as indicated by recent fisheries and dFAD buoy density data (Lopez et al., 2020, 2021, 2022). French Polynesia, however, located directly west of this area, experiences significant dFAD stranding rates from dFADs deployed in the EPO (Escalle, Mourot, et al., 2022), highlighting the likely high rate of dFAD loss in the southern EPO. Moderate to low connectivity of vFADs was also simulated between the equatorial deployment regions and nesting habitats of both species near Mexico and Costa Rica–Nicaragua (i.e., up to 1.7% in scenario 2 and 23% in scenario 1). Purse‐seine fishers mentioned that sea turtle sightings and interactions were frequent in this area and indicated the possibility of certain dFADs drifting east and stranding along the Central American coastline (Figure 6). Several important nesting habitats for both the hawksbill and leatherback turtles occur on the Central American coast. The dFAD strandings in this area could therefore interfere with the nesting or early development of sea turtles (Fujisaki & Lamont, 2016; Laurance et al., 2008).

The Pacific leatherback sea turtle nesting populations on both sides of the Pacific have declined by more than 80% over the last three generations and are at risk of extinction (NOAA Fisheries, 2022b); hence, the need to conserve as many individuals and increase hatchling outputs (Laud OPO Network, 2020). Regarding the overlap between dFAD aggregation and leatherback foraging habitats, although potential interactions seem to be low, considering the population status of leatherbacks, any entanglements, especially lethal drownings, would have a large impact. Determining rates of ghost fishing entanglements with dFADs in‐ and outside fishing grounds will require directed research expeditions inspecting the full depth of dFAD appendages.

The hawksbill sea turtle is also a critically endangered species, with population declines throughout its range. It, however, has the advantage of more nesting areas across the Pacific Ocean, but most of these distinct areas constitute <100 nests/year (Gaos et al., 2017; SWOT, 2008). The largest nesting area is in Solomon Islands, with less than 400 nests/year documented between 1991 and 2012 (Hamilton et al., 2016; Mortimer, 2002). Moderate connectivity of vFADs from the WPCO was seen for this nesting region (maximum of 4.6% from scenario 2). Although the impact of stranded dFADs is unknown, other large marine debris deter the nesting of sea turtles or reduce hatchling survival (Fujisaki & Lamont, 2016; Laurance et al., 2008).

### Connectivity of pacific gyres

Interestingly, the simulations showed little connectivity between the equatorial Pacific and the north Pacific gyre (also referred to as the Great Pacific Garbage Patch, 25°N−40°N and 130°W−160°W) (Figures 3 & 4). This oceanic convergence zone entrains floating marine debris at higher concentrations than outside the gyre (Lebreton et al., 2018). The lack of simulated connectivity may in part be explained by the assumption that the 50‐m‐deep appendages of dFADs remain intact during the 24 months after deployment. In reality, dFADs lose all or a portion of their appendage after some unknown time. The shallower floating debris, being more affected by wind and waves, would have a different drift trajectory and speed (Imzilen et al., 2018; Scutt Phillips et al., 2019), possibly resulting in greater dFAD accumulation in the North Pacific gyre. Further simulations examining alternate depth profiles and extended drift times may reveal stronger connectivity of dFADs in the Great Pacific Garbage Patch.

On the contrary, a large portion of vFADs from the EPO were simulated to drift toward the southern Pacific gyre, where a much greater vFAD accumulation took place. This matches the observed drift patterns identified by fishers, where dFADs trajectories in the southeast Pacific experienced fewer eddy structures and eastward flowing counter currents than in the north.

### Comparison to fishers’ knowledge

Overall, tuna purse‐seine fishers’ knowledge of dFAD trajectories significantly overlapped with our vFAD particle simulations and provided information to assist with future dFAD modeling efforts. The main tendency of dFADs to drift west was well described by fishers. Densities were high along the coast of PNG and Solomon Islands, as was accumulation of dFADs in the South Pacific gyre. Similarly, purse‐seine vessels and sea turtle interactions described by fishers somewhat matched the areas of leatherback and hawksbill turtles’ oceanic and coastal habitats we identified. Where they differed were in regions outside the fishing grounds (absent on the fishers’ map) or regions where other sea turtle species occur (present on the fishers’ map) because these species (e.g., green, olive ridley, and loggerhead turtles) more often interact with the purse‐seine fishery (IATTC, 2023a; Peatman et al., 2018).

Beyond the fishers’ geographical knowledge, especially outside the equatorial fishing grounds, there were few data to compare to our model results. One growing source of information is the dFAD's buoys geolocation data that are being submitted to regional management bodies and research institutes (Escalle, et al., 2023; IATTC, 2023b). However, they are very limited outside fishing grounds because buoys are commonly deactivated. Alternatively, data collected in coastal areas regarding stranding events of dFADs and the potential ecosystem impacts they may cause (Escalle, et al., 2022) could also be used to inform model results. Eleven percent of dFADs end up stranded in the WCPO, with higher numbers in PNG and Solomon Islands (Escalle et al., 2019), and French Polynesia receives more than 1000 dFADs stranding per year (Escalle, et al., 2022). Most countries and territories located in the WCPO purse‐seine fishing grounds also presented some levels of dFAD stranding events (e.g., Nauru, Tuvalu, Kiribati, the Federated States of Micronesia) (Escalle et al., 2019; Escalle, et al., 2022). Numbers were lower in the Hawaiian Islands and Palmyra Atoll (Donohue, 2005; Royer et al., 2023). Escalle et al. (2019) showed the importance of local‐scale ocean dynamics on the actual levels of stranding on coastlines in the Pacific, despite large‐scale ocean processes being responsible for the distribution of dFADs while drifting.

### dFAD deployment and design implications

In traditional dFAD structures, old purse‐seiner synthetic netting is reused as the main component to construct the ∼50‐m submerged tail and to cover the surface components of the raft (Escalle, et al., 2023). In the Pacific Ocean, RFMOs have implemented measures to mitigate the ecosystems impacts of dFADs. For instance, the use of netting in dFADs construction will be prohibited after January 2024 in the WCPO (WCPFC CMM‐23‐01) and after 2025 in the EPO (IATTC C‐23‐04). Before these measures are fully implemented, the use of low‐entanglement‐risk dFADs, specifically employing netting with small mesh size or wrapped into bundles, is permitted (ISSF, 2019). However, these designs eventually degrade creating larger holes or unwrapping mesh; thus, they pose a high risk of entanglement. Therefore, we stress the importance of implementing fully nonentangling dFADs as soon as possible to reduce negative impacts on declining sea turtle populations. This will help mitigate and reduce dFAD entanglement risk with sea turtles and other vulnerable species, such as sharks, in oceanic and coastal areas.

Furthermore, RFMOs encourage fishing fleets to transition to biodegradable dFADs, which are composed of organic materials that naturally degrade after their operational lifespan (Zudaire et al., 2023). In the EPO, a stepwise timeline is in place to transition to 100% biodegradable dFADs by 2030 (IATTC C‐23‐04). Recent research on non‐entangling and biodegradable dFADs tested new designs, such as the jelly‐FAD, that drift slowly and remain in working condition for the duration required by fishers (Moreno et al., 2022). If only non‐entangling and biodegradable dFADs were used, they may degrade before reaching some essential coastal habitats for sea turtles located outside the fishing grounds and, therefore, reduce the impact of turtle entanglement and habitat damage. However, our results indicated that nonentangling and biodegradable dFADs could still arrive in TZs in the fishing grounds (e.g., SB, PNG, EP1, and EP2). Although the lack of netting should reduce entanglement risks, other management options could therefore also be considered to mitigate potential impacts in these areas. This could include reduced dFAD deployment limits, spatial or temporal closures, spatial or temporal regulations on deployment, or dFAD retrieval plans. All these management measures include a spatial component, which could be guided by our results or similar simulation experiments with effect on connectivity and density of dFADs further quantified by examining real and simulated trajectories.

Current operational patterns appear to result in a great density of dFADs being deployed south of the equator, which reduces the interaction and connectivity between vFADs and some of our identified sea turtle habitats. However, a northern or more homogenic shift in dFAD deployment strategies could lead to higher dFADs arrival in many important sea turtle habitats. Near‐real‐time changes in dFAD fishing strategies by fleets should be carefully monitored to adapt management measures if necessary.

### Future research

Further validation of our results should be undertaken with real dFAD trajectory data where possible. Imzilen et al. (2018) conducted a comparative analysis in the Atlantic and Indian oceans, demonstrating the Lagrangian model's favorable predictive performance in representing mean dFAD densities at the basin scale in both regions. This positive outcome suggests that Lagrangian model could also be valid to accurately represent dFAD densities in the Pacific Ocean. In the WCPO, observed dFAD densities have been compared with simulated dFAD densities with Lagrangian simulation based on ocean currents from the top 50 m and also showed similar results (Scutt Phillips et al., 2019). However, one of the constraints is the limited availability of real dFAD trajectories. The dFAD data owners, the fishing companies, usually try to keep these tracking data private for competitive reasons. However, such data, often partial trajectories, exist within buoy manufacturing companies, and some dFAD trajectories are being made available, with a time lag, for scientific studies (Escalle, et al., 2023; IATTC, 2023b). Another valuable source of information on dFAD fate is fishers’ knowledge. They not only have considerable experience on dFAD trajectories, but they can also improve understanding of how and why adaptive alterations in fishing strategies may take place (Moreno, et al., 2007; Moreno, et al., 2007; Murua et al., 2023). Such fisher‐derived information can help create more accurate model algorithms or management options that incorporate adaptive dFAD fishing tactics.

Although operational dFADs typically last up to 1 year, the precise lifespan and rate of decay of dFADs under various construction designs and ocean conditions remain uncertain. The impact of this decay on simulated spatial density could be examined by using a survival probability function through time (Scutt Phillips et al., 2019). However, given the lack of knowledge on long‐term breakdown of rafts, more information is required to model the process of decay accurately. Scenario testing of alternative dFAD designs with differing lifespans, using simulation modeling, would reveal whether large‐scale changes in spatial distribution are likely.

Research into the fate of sea turtles that interact with dFADs, through sea turtle satellite tagging programs for instance, would help evaluate the real impact of those interactions (i.e., do sea turtles approach and get entangled). Although fisheries observer data are available, dFADs are rarely visited and observers have limited ability to identify underwater entanglements from the vessel's deck. Research cruises investigating active and lost dFADs and sea turtle entanglement rates (Filmalter et al., 2013) could therefore be employed.

To better understand the overlap of dFAD trajectories with sea turtle movements at sea, simulated dFAD trajectories and aggregation areas could be compared with real sea turtle movements (Liang et al., 2023), through existing tagging data (http://www.seaturtle.org/tracking/explorer/#) or even using species distribution models (Gaspar & Lalire, 2017). For instance, telemetry and other fine‐ and large‐scale sea turtle distribution data should be examined to better assess the degree of overlap in the areas explored in this study. In addition, incorporating all sea turtle species in future studies, along with comparing the model to turtle sightings, entanglement events, and catches, would offer a more comprehensive understanding of the impact of dFADs on turtle populations.

Although our results indicate that dFADs deployed uniformly in equatorial tuna purse‐seine fishing grounds overlap with important sea turtle migratory routes and coastal habitats, this potential interaction is reduced when observed dFAD deployment and high dFAD density are taken into consideration. Additional research is needed to quantify more accurately how sea turtles are affected by dFADs, particularly in the open ocean, on coral reef foraging grounds, and at nesting beaches. Lagrangian simulations provide a useful tool to assess the connectivity between some pelagic and coastal zones and key dFAD areas, which can inform the development of potential effective mitigation strategies. However, the extent of actual dFAD stranding events, and their ecological impacts, cannot be fully determined in this way. Working with real dFAD trajectories and collecting additional in situ data to quantify the number and consequences of stranding events should be therefore a priority. The use of fully non‐entangling dFADs constructed with biodegradable materials may reduce sea turtle entanglement and other ecosystem impacts when stranding. These and other mitigation alternatives can be incorporated into best practice guidelines or management options adopted by tuna RFMOs to diminish undesirable impacts on sea turtles and other vulnerable species in the Pacific and other oceans.

## AUTHOR CONTRIBUTIONS

Lauriane Escalle, Joe Scutt Phillips, Jon Lopez, Jennifer M. Lynch, Hilario Murua, Sarah‐Jeanne Royer, Yonat Swimmer, and Gala Moreno designed and interpreted the analyses. Lauriane Escalle and Joe Scutt Phillips performed the analyses. Jefferson Murua, Gala Moreno, Hilario Murua, Jon Lopez, and Lauriane Escalle participated in the fishers’ workshops. Alex Sen Gupta provided advice and support for the Lagrangian simulations. All authors contributed to the writing and the revising of the manuscript.

## Supporting information

Supplementary materials

Supplementary materials

Supplementary materials

Supplementary materials
