# Supplementary figures and images for: Simulating drifting fish aggregating device trajectories to identify potential interactions with endangered sea turtles
Source: Conserv Biol. 2024 May 20;38(6):e14295. doi: 10.1111/cobi.14295 (PMC11589028; doi:10.1111/cobi.14295)

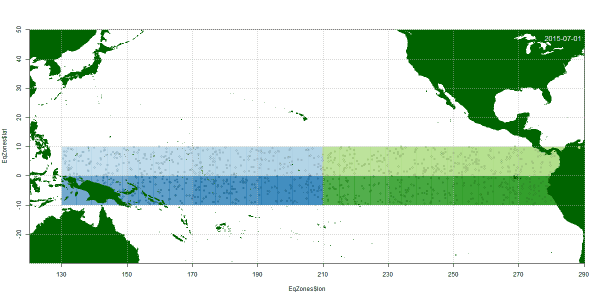

Supplement: Supplementary file 1 — Supplementary materials [file COBI-38-e14295-s004.gif]

< 3 months

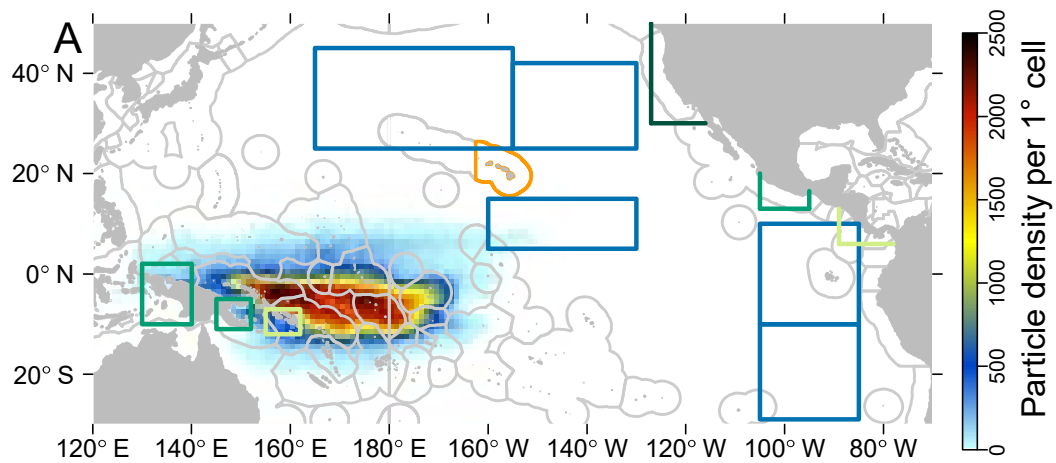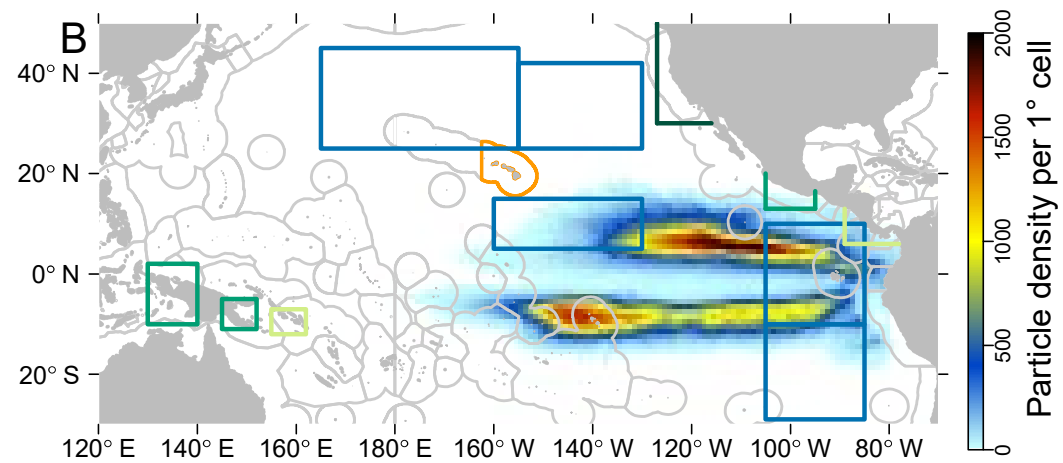

3-12 months

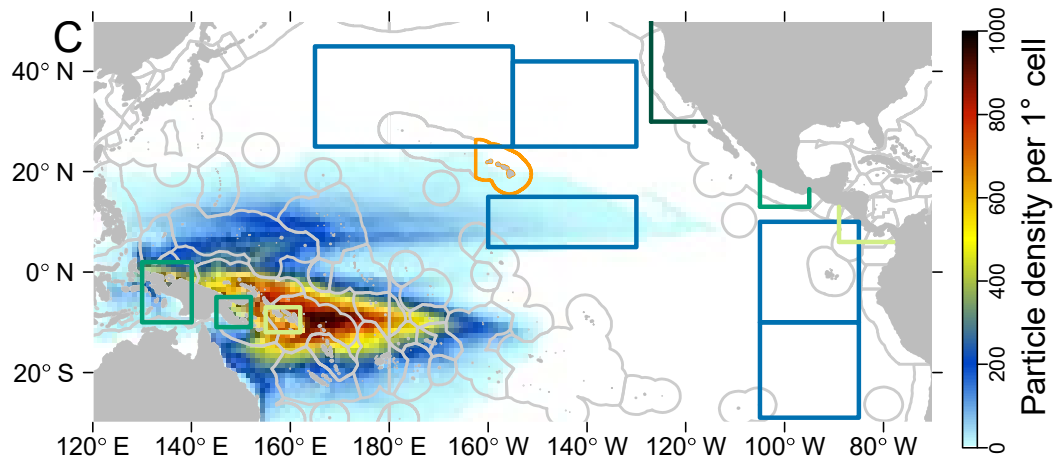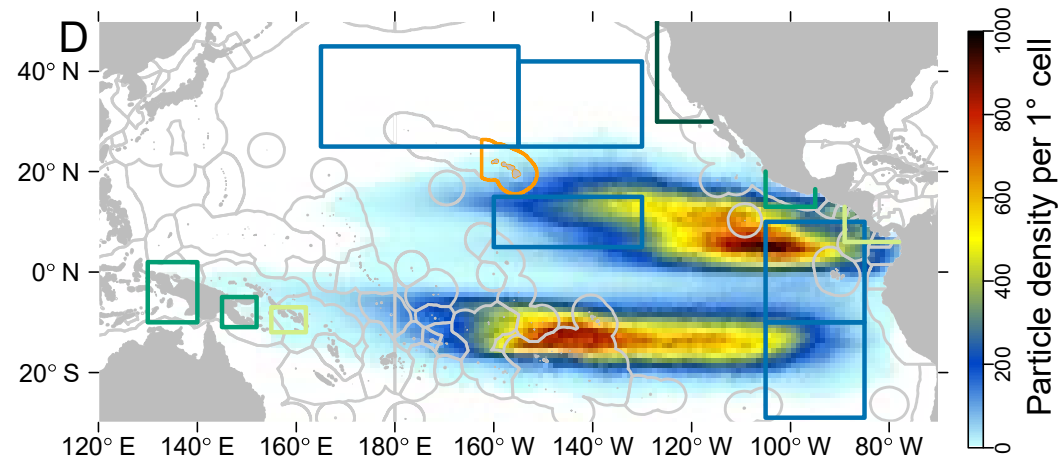

12-24 months

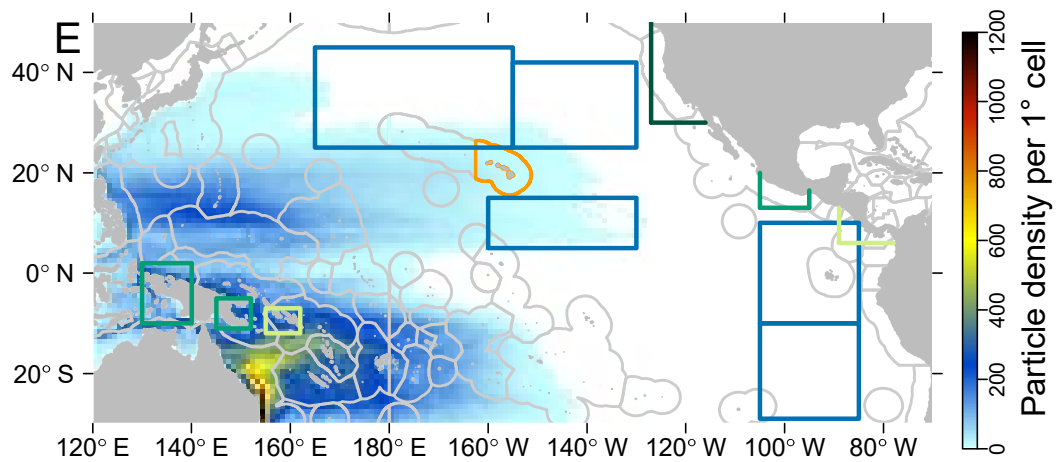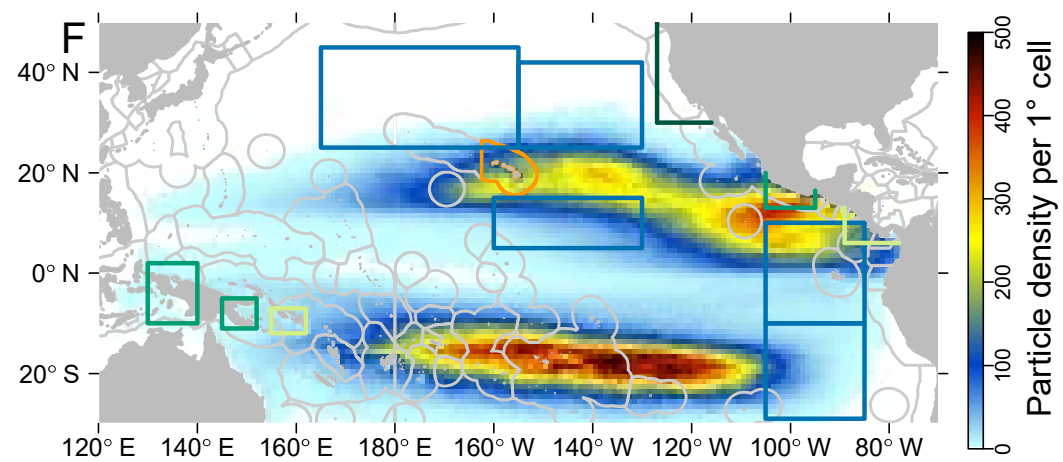

Supplement: Supplementary file 3 — Supplementary materials [file COBI-38-e14295-s002.pdf]
