# Supplementary material for: Simulating drifting fish aggregating device trajectories to identify potential interactions with endangered sea turtles
Source: Conserv Biol. 2024 May 20;38(6):e14295. doi: 10.1111/cobi.14295 (PMC11589028; doi:10.1111/cobi.14295)
Supplement: Supplementary file 2 — Supplementary materials [file COBI-38-e14295-s003.pdf]

	TZ	Other			KE 1			KE 2			CCE			IND			PNG			SB			EEP			MX			CR−NG			EP 1			EP 2			MHI		
	Months	3	12	24	3	12	24	3	12	24	3	12	24	3	12	24	3	12	24	3	12	24	3	12	24	3	12	24	3	12	24	3	12	24	3	12	24	3	12	24	3	12	24
EZ																																											
WCPO	1	95.8	96.1	90.5	0	0.3	5.6	0	0	0.1	0	0	0	4.1	3	2.5	0	0.2	0.4	0	0.2	0.3	0	0.1	0.5	0	0	0	0	0	0	0	0	0	0	0	0	0	0.1	0.2
	2	98.1	95.9	90	0	0.3	6.2	0	0	0.8	0	0	0	0.4	1.1	1	0.1	0.5	0.6	0.2	0.5	0.4	1.1	0.8	0.3	0	0	0	0	0	0	0	0	0	0	0	0	0	0.8	0.8
	3	87.4	92.4	85.7	0	1	8.1	0	0.1	2.5	0	0	0	0	0.5	0.5	0	0.3	0.3	0.1	0.3	0.2	12.4	2.5	0.1	0	0	0.1	0	0	0	0	0	0	0	0	0	0	2.9	2.3
	4	63.8	85.7	79	0	1.3	8.3	0	0.7	6.1	0	0	0	0	0.3	0.3	0	0.2	0.2	0	0.2	0.2	36	4	0.4	0	0.4	0.5	0	0	0.1	0	0.6	0.5	0	0	0	0.2	6.5	4.3
	9	66.6	75.9	77.7	0	0	1	0	0	0	0	0	0	26.2	18.1	14.2	6	4.8	5.3	1.1	1.1	0.9	0	0	0.6	0	0	0	0	0	0	0	0	0	0	0	0	0	0	0.1
	10	80	80.5	80.8	0	0	0.4	0	0	0	0	0	0	3.2	6.4	6.1	6.2	7	7.4	10.6	6	4.8	0	0.2	0.4	0	0	0	0	0	0	0	0	0	0	0	0	0	0	0.1
	11	97.6	88.9	88.5	0	0	0.4	0	0	0.1	0	0	0	0.2	3.9	4	0.3	2.9	3.4	1.7	3.9	3.1	0.1	0.3	0.3	0	0	0	0	0	0	0	0	0	0	0	0	0	0.1	0.2
	12	99.2	93.9	92.7	0	0	0.3	0	0	0.1	0	0	0	0	2	2.3	0	1.4	2	0	2.2	2.2	0.8	0.3	0.2	0	0	0	0	0	0	0	0	0	0	0	0	0	0.2	0.2
EPO	5	49.3	75.4	73.3	0	0.3	4.7	0	1.3	9.8	0	0	0	0	0.2	0.2	0	0.1	0.1	0	0.2	0.1	50	8.5	1	0	0.8	1.4	0	0.3	0.4	0.4	3.4	1.8	0	0	0.1	0.2	9.6	7.1
	6	74.3	73.6	74.8	0	0	1.2	0	0.6	7.1	0	0	0	0	0	0.1	0	0	0.1	0	0.1	0.1	12.4	9.5	2	0.4	2.1	3.2	0.1	1.3	0.9	12.9	9.9	4.8	0	0.1	0.3	0	2.7	5.5
	7	55.6	65.6	75.4	0	0	0.2	0	0	1.6	0	0	0	0	0	0	0	0	0	0	0	0	0.8	6	2.7	3.3	6.2	5.9	5	3.5	1.9	35.2	17.7	8.8	0	0.8	1	0	0.2	2.4
	8	39.9	59.6	74.6	0	0	0.1	0	0	0.3	0	0	0	0	0	0	0	0	0	0	0	0	0	2.4	2.6	1.7	10.2	7.1	22.5	5.6	2.4	35.8	18.2	9.9	0	3.9	2	0	0	0.9
	13	98.6	97.6	96.6	0	0	0.2	0	0	0.2	0	0	0	0	0.5	0.8	0	0.4	0.7	0	0.8	1.1	1.3	0.4	0.1	0	0	0	0	0	0	0.1	0.1	0	0	0	0	0	0.2	0.2
	14	98.3	98.9	98.7	0	0	0	0	0	0.1	0	0	0	0	0	0.2	0	0	0.2	0	0.1	0.3	0.4	0.4	0.1	0	0.1	0.1	0	0	0	1.3	0.3	0.2	0	0.1	0	0	0.1	0.2
	15	60.6	92.8	97.9	0	0	0	0	0	0.1	0	0	0	0	0	0	0	0	0	0	0	0.1	0	0.2	0.1	0	0.1	0.1	0.1	0	0	18.9	0.4	0.2	20.3	6.4	1.4	0	0	0.1
	16	20.2	38.8	80.7	0	0	0	0	0	0	0	0	0	0	0	0	0	0	0	0	0	0	0	0.4	0.2	0	0.2	0.3	0.3	0.2	0.1	52.1	2.6	0.6	27.4	57.9	18	0	0	0.1
