# Supplementary material for: Simulating drifting fish aggregating device trajectories to identify potential interactions with endangered sea turtles
Source: Conserv Biol. 2024 May 20;38(6):e14295. doi: 10.1111/cobi.14295 (PMC11589028; doi:10.1111/cobi.14295)
Supplement: Supplementary file 4 — Supplementary materials [file COBI-38-e14295-s001.docx]

**Supplementary materials**

Escalle et al., (2024) Simulating drifting fish aggregating device trajectories to identify potential interactions with endangered sea turtles. Conservation Biology

**Appendix S1.** Animation displaying the drift patterns of the simulated vFADs under the simulation framework described in this paper: <https://zenodo.org/doi/10.5281/zenodo.10815559>

**Appendix S2.** Percent connectivity matrix of virtual fish aggregating device (vFAD) particles in the time-forward simulation during the three El Niño–Southern Oscillation periods considered combined from scenario 1 in which vFADs were evenly seeded in equatorial zones (EZ) (rows) and arrived in sea turtle habitat (TZ) (columns) within 3, 12, or 24 months (sub columns) (cell colors, proportion of simulated particles arriving in each TZ by drift time increases with increasing intensity; other, locations outside the specified TZ; WCPO, Western and Central Pacific Ocean; EPO, Eastern Pacific Ocean).

**Appendix S3.** Time-integrated spatial probability density for virtual particles (vFADs) deployed in Scenario 2a, evenly across dFAD deployment hotspots in the WCPO (left panels A, C and E; *EZ*s 1–4 and 9–12) and the EPO (right panels B, D and F; *EZ*s 5–8 and 13–16) during the three ENSO periods considered combined and over three drifting periods after deployment.
